# Supplementary material for: Complications of biliary stenting versus T-tube insertion after common bile duct exploration: A systematic review and meta-analysis
Source: PLoS One. 2023 Jan 20;18(1):e0280755. doi: 10.1371/journal.pone.0280755 (PMC9858848; doi:10.1371/journal.pone.0280755)
Supplement: Attachment — Submitted filename: Review report_TuoYang.docx [file pone.0280755.s002.docx]

This research article by Rudiman et al compared post T-tube or stent insertion with respect to procedure complications, efficacies and efficiencies. The authors analyzed 534 biliary stents and 546 T-tube patients and results showed that stenting after common bile duct exploration (CBDE) is superior to T-tube insertion with shorter operation time and hospital state as well as lower overall postoperative complication rate.

I believe that this manuscript is suitable for publication in Plos One. However, there are still some minor points that the authors need to address

1. all figures appear to be very blurry. Please upload high res figures.

2. It would be very informative to include more background information of stent and T-tube insertions in general.

2. Some sentences need to be described more clearly, for example, Page 16, “Thus, indicating no publication bias in this study…”, there was no subject in this sentence.
