# Supplementary material for: Complications of biliary stenting versus T-tube insertion after common bile duct exploration: A systematic review and meta-analysis
Source: PLoS One. 2023 Jan 20;18(1):e0280755. doi: 10.1371/journal.pone.0280755 (PMC9858848; doi:10.1371/journal.pone.0280755)
Supplement: Attachment — Submitted filename: Response to Reviewers.docx [file pone.0280755.s003.docx]

Dear Editor-in-Chief of *PLoS ONE*,

Thank you for allowing us to submit a revised draft of our manuscript. We appreciate the time and effort you and the reviewers have dedicated to providing your valuable feedback on our manuscript. We are grateful to the reviewers for their insightful comments on our paper. We have been able to incorporate changes to reflect most of the suggestions provided by the reviewers.

Here is a point-by-point response to the reviewers’ comments and concerns.

Reviewer 1

Author reviewed and did meta-analysis of 16 papers. These papers are published between 2004 – 2022 include 1,080 patients' (534 biliary stents and 546 T-tube) outcome of biliary stenting or T-tube insertion after common bile duct exploration operation. Author statistically analyzed the major complications rates, operation time, hospital stay length, etc. And author concluded stenting as biliary drainage after CBDE was superior to T-tube insertion.
Although the manuscript is drafted well and technically sounded good. The two candidates are just not comparable. Modern surgery are tend to be as less invasive as possible. While author also mentioned T-tube insertion already been dropped off by many places in the world and sooner or later it will not be used at all. On the other hand, the developing of stenting technology never stopped, stent itself even can be drug loaded or biodegradable.

**Author’s comment:** *Thank you for your insightful comment. We agree that a less invasive approach has been a favorable technique in modern surgery. T-tube should be dropped off and shifted to minimally invasive method. Through this study, we would like to emphasize to the readers that biliary stent should be the preferred method due to its superiority in several aspects (shorter operation time, shorter hospital stay, and lower overall complications rate). The development of biliary stents has been quite remarkable in the last decade; however, these two candidates are feasible to be compared because in the last five years, our included studies still compared both methods and the limitation of healthcare services in developing countries force surgeons to choose T-tube as the initial option. With all deliberation, T-tube remains a realistic possibility without increasing patients’ mortality and morbidity in developing countries.*

*We also incorporated further reasons on writing this manuscript in the Discussion section.*

Reviewer 2

1. All figures appear to be very blurry. Please upload high res figures.

**Author’s comment:** *We have updated all figures using Prelight Analysis and Conversion Engine (PACE) digital diagnostic tool to ensure all figures meet PLOS requirements.*

2. It would be very informative to include more background information of stent and T-tube insertions in general.

**Author’s comment:** *Thank you for your consideration. In the Introduction section, we have incorporated additional background information on biliary stent and T-tube.*

3. Some sentences need to be described more clearly, for example, Page 16, “Thus, indicating no publication bias in this study…”, there was no subject in this sentence.

**Author’s comment:** *Thank you for your valuable feedback. We have completed that sentence.*

We look forward to hearing from you in due time regarding the submission, responses, further questions, and comments you may have.

Sincerely,

Reno Rudiman
