# Supplementary material for: Complications of biliary stenting versus T-tube insertion after common bile duct exploration: A systematic review and meta-analysis
Source: PLoS One. 2023 Jan 20;18(1):e0280755. doi: 10.1371/journal.pone.0280755 (PMC9858848; doi:10.1371/journal.pone.0280755)
Supplement: Attachment — Submitted filename: Response to Reviewer.docx [file pone.0280755.s004.docx]

Dear Editor-in-Chief of *PLoS ONE*,

Thank you for allowing us to submit a revised draft of our manuscript. We appreciate the time and effort you and the reviewers have dedicated to providing your valuable feedback on our manuscript. We are grateful to the reviewers for their insightful comments on our paper. We have been able to incorporate changes to reflect most of the suggestions provided by the reviewers.

Here is a point-by-point response to the reviewer’ comments and concerns.

Reviewer 1

Reviewer #1: In this revision, author added more back ground information about T-tube insertion procedure and author also added explanation about how T- tube insertion compare to biliary stenting in discussion section. Manuscript is getting better but there are still have few more things I think author should make it more clear: Author referred PRISMA guideline but Review Manager software has been used for conducting meta-analysis. Therefore, beside "The PRISMA flowchart of the studies selection process", how the Review Manager been configured and handled should be described in details since most of the data are generated by the Review Manager software in this paper. Also, since this is a scientific paper doing meta-analysis. Statistical analysis in "Method" section should be explained with a lot more details. The manuscript mentioned 4 formulas in total. Please elaborate: Is there any result from PICOS formula? What is Mantel-Haenszel formula with random-effect models to calculate RR and CI? And what is Inverse-Variance formula with random-effect models that was used to assess the SMD and SD? Although formula to convert means and standard deviations (SD) for meta-analysis data synthesis has a reference. How this formular been used in this manuscript needs more explanation.

**Author’s comment:** Thank you for your insightful suggestion.

1. Yes, there is a result from the PICOS formula, which we have elaborated in the Result Section and all the outcomes were statistically analysed

2. We have described further the Mantel-Haenszel and Inverse-Variance formula in the Synthesis Methods Section.

We look forward to hearing from you regarding the submission, responses, questions, and comments you may have.

Sincerely,

Reno Rudiman
